# Structural and mechanistic study of a novel inhibitor analogue of *M. tuberculosis* cytochrome bc_1_:aa_3_

**DOI:** 10.1038/s44386-025-00008-3

**Published:** 2025-04-02

**Authors:** Amit K. Verma, Robbert Q. Kim, Dirk A. Lamprecht, Clara Aguilar-Pérez, Sarah Wong, Nicolas Veziris, Alexandra Aubry, José M. Bartolomé-Nebreda, Rodrigo J. Carbajo, Jennefer Wetzel, Meindert H. Lamers

**Affiliations:** 1 Department of Cell and Chemical Biology, Leiden University Medical Center, Einthovenweg 20, 2333 ZC Leiden, The Netherlands; 2 Janssen Pharmaceutica, Global Public Health, Turnhoutseweg 30, 2340 Beerse, Belgium; 3 Sorbonne Université, INSERM, Centre d’Immunologie et des Maladies Infectieuses, U1135, AP-HP. Sorbonne-Université, Fédération de Bactériologie, Centre National de Référence des Mycobactéries et de la Résistance des Mycobactéries aux Antituberculeux, Paris, France; 4 Global Discovery Chemistry, Janssen-Cilag, S.A., a Johnson & Johnson Innovative Medicine company, c/ Jarama, 75 A, 45007 Toledo, Spain; 5 In Silico Discovery, Janssen-Cilag, S.A., a Johnson & Johnson Innovative Medicine Company, c/ Jarama, 75A, 45007 Toledo, Spain

**Keywords:** Drug discovery, Target validation

## Abstract

Drug-resistant tuberculosis (TB) continues to challenge treatment options, necessitating the exploration of new compounds of novel targets. The mycobacterial respiratory complex cytochrome bc_1_:aa_3_ has emerged as a promising target, exemplified by the success of first-in-class inhibitor Q203 in phase 2 clinical trials. However, to fully exploit the potential of this target and to identify the best-in-class inhibitor more compounds need evaluation. Here, we introduce JNJ-2901, a novel Q203 analogue, that demonstrates activity against multidrug-resistant *M. tuberculosis* clinical strains at sub-nanomolar concentration and 4-log reduction in bacterial burden in a mouse model of TB infection. Inhibitory studies on purified enzymes validate the nanomolar inhibitions observed in mycobacterial cells. Additionally, cryo-EM structure analysis of cytochrome bc_1_:aa_3_ bound to JNJ-2901 reveals the binding pocket at the menaquinol oxidation site (Qp), akin to other substate analogue inhibitors like Q203 and TB47. Validation of the binding site is further achieved by generating and isolating the JNJ-2901 resistant mutations in *M. tuberculosis*, followed by purification and resistance analysis of the resistant cytochrome bc_1_:aa_3_ complex. Our comprehensive work lays the foundation for further clinical validations of JNJ-2901.

## Introduction

Tuberculosis (TB), caused by *Mycobacterium tuberculosis*, remains a significant threat to global health, with over 10.6 million individuals falling ill and more than 1.3 million deaths recorded in 2022^1^, underscoring its profound impact on global health. The prevalence of drug-resistant (MDR) and extensively drug-resistant (XDR) TB necessitates longer treatment durations (18-20 months) with multiple drugs and often leads to poor treatment outcomes compared to drug-sensitive TB cases. Despite recent MDR/XDR-TB treatment breakthroughs, such as the use of bedaquiline, pretomanid, and linezolid with or without moxifloxacin (BPaL/BPaLM)^2^, the emergence of bedaquiline-resistant *M. tuberculosis* strains could pose a threat to successful treatment outcomes^3–6^. There is an urgent need for the development of new therapies and strategies to combat the escalating challenge that requires the identification of novel compounds and targets along with a thorough understanding of their modes of action and resistance development.

Bedaquiline is a key component of the new TB treatment regimens currently undergoing development^7–10^. It inhibits the F_1_F_0_ ATP synthase enzyme of the mycobacterial electron transport chain^11–13^. The discovery of bedaquiline has sparked interest in other enzymes of the electron transport chain as novel targets for TB drug discovery^14–16^. Among these targets, the cytochrome bc_1_:aa_3_ enzyme complex (cytochrome bc) stands out, as evidenced by the progress of its inhibitors like Q203^17^ and TB47^18^. Q203 has shown to be safe in a phase 1b clinical trial^19^ and shown good results in the phase 2 clinical trials^20^, while TB47 is in preclinical stage (https://www.newtbdrugs.org/pipeline/clinical). Cytochrome bc is an intermediate complex in the electron transport chain and functions as a terminal electron acceptor, converting oxygen to water and contributing to the proton motive force, required for ATP generation for bacterial survival^21–23^. Both the molecules Q203 and TB47 are substrate analogs (menaquinone) and function by preventing substrate binding^24,25^. Another line of evidence supporting cytochrome bc as a promising drug target was found in a genome-wide CRISPRi screen in *M. tuberculosis*, where it was found to be one of the top vulnerable targets^26^.

Considering the high attrition rate of the drug discovery process and to increase the chance of bringing cytochrome bc inhibitors to the treatment stage, additional inhibitors must be added to the pipeline, and consequently, multiple efforts are made to search for novel inhibitors of cytochrome bc, see^27–30^ and further work reviewed in Wani et al.^31^ and Bajeli et al.^32^. Here, we introduce JNJ-2901, a novel analog of Q203 and inhibitor of cytochrome bc. We show that JNJ-2901 inhibits the growth of clinical multidrug-resistant *M. tuberculosis* strains at sub-nanomolar concentrations and reduces lung colony forming unit (CFU) by 4 logs in a mouse model of *M. tuberculosis* infection. At the enzymatic level, we confirm JNJ-2901’s nano-molar inhibitory potency. By determining the cryo-EM structure of a modified cytochrome bc from *M. smegmatis* in which the active site has been made identical to that of *M. tuberculosis* we show that JNJ-2901 occupies the menaquinol oxidation site (Qp) in a manner analogous to Q203 and TB47. Furthermore, we generated laboratory mutants of JNJ-2901 in *M. tuberculosis* and identified the resistant mutations within the binding pocket. To gain further insight into the resistant mechanism, we purified the resistant enzyme complex and analyzed the resistance at the enzymatic level. These resistant enzymes will serve as tools to screen novel inhibitors targeting resistant enzyme complexes. Our comprehensive work establishes that JNJ-2901 is an effective, potent inhibitor of *M. tuberculosis* cytochrome bc with the potential for further development towards a novel antibiotic in the continuing battle against drug-resistant TB.

## Results

### JNJ-2901 inhibits bacterial growth in-vitro and in-vivo

**Fig. 1:**
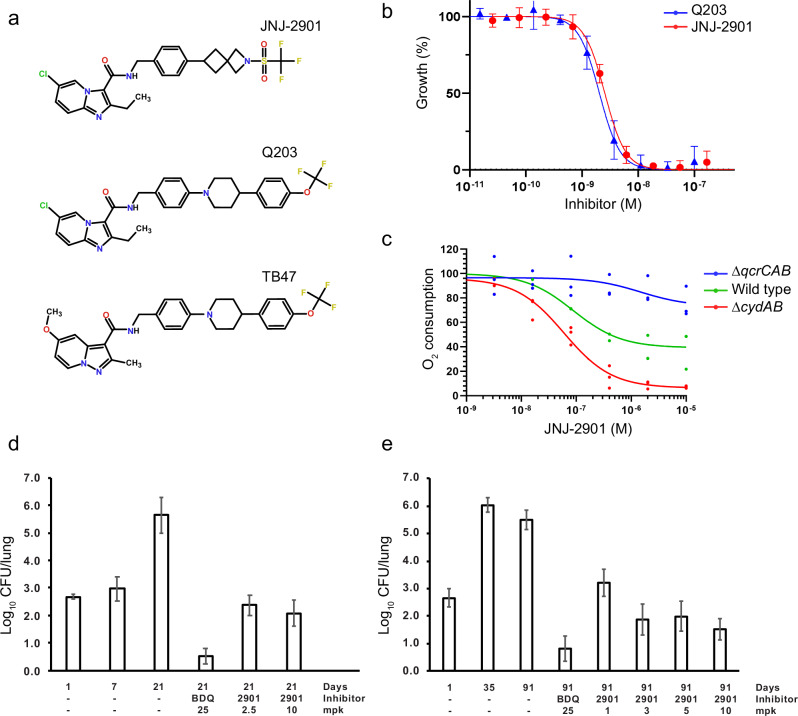
Characterization of a novel mycobacterial cytochrome bc inhibitor. **a** Chemical structures of JNJ-2901, Q203, and TB47. **b** Inhibitory profile of Q203 and JNJ-2901 on *M. tuberculosis* H37Rv-Δ*cydAB* grown in 7H9 medium. MIC_50_ values Q203: 2.0 ± 0.1, JNJ-2901: 2.5 ± 0.1. **c** Inhibitory activity of JNJ-2901 on membranes prepared from *M. smegmatis* wild-type strain, a cytochrome bc knockout strain (Δ*qcrCAB*) or a cytochrome bd knockout strain (Δ*cydAB*). **d** Survival of Mtb H37Rv cytochrome bd knockout strain (Δ*cydAB*) in an acute mouse model upon exposure with bedaquiline (BDQ) or JNJ-2901 (mpk = milligram per kilogram). Each group contained 6 mice, and error bars indicate standard deviation. **e** Survival of Mtb H37Rv-Δ*cydAB* in a chronic mouse model upon exposure with bedaquiline (BDQ) or JNJ-2901 (mpk = milligram per kilogram).

JNJ-2901 is a novel analog of the *M. tuberculosis* cytochrome bc inhibitor Q203^17^ (also known as Telacebec) and TB47^18^ (Fig. 1a). To measure its potential in reducing bacterial growth, we tested JNJ-2901 on a panel of 18 clinical isolates of *M. tuberculosis* with different antibiotic resistance profiles for commonly used antibiotics (Table 1). For all 18 clinical strains, we found that JNJ-2901 is a potent inhibitor of growth in the Middlebrook 7H9 medium with a MIC of ≤0.5 μg/L (≤1 nM). Interestingly, the H37Rv and Erdman reference strains are less sensitive to JNJ-2901 (MIC of >8 μg/L, >16 nM). The reduced susceptibility of H37Rv for inhibition of cytochrome bc was also observed by others^26,33,34^. Furthermore, it was shown that in this strain, the deletion or inhibition of the cytochrome bd oxidase results in restoration of cytochrome bc sensitivity^33–35^, suggesting that cytochrome bd could take over the bulk transport of protons when cytochrome bc is inhibited. Also in our hands, we find that a deletion strain for cytochrome bd, *M. tuberculosis* H37Rv (H37Rv-Δ*cydAB*), shows restored sensitivity for JNJ-2901, with an MIC_50_ of 2.5 ± 0.1 nM, similar to Q203 (MIC_50_ of 2.0 ± 0.1 nM) (Fig. 1b).

**Table 1:** MIC^1^ values for JNJ-2901 on two reference strains and 18 multi-drug-resistant clinical strains with different antibiotic resistance profiles

Strain	MIC^a^ (μg/L)	Resistance profile
H37Rv Pasteur	>8	n.a.
Erdman	>8	n.a.
661706043123	≤0.5	INH, PAS
661710016056	≤0.5	INH, S, PAS
661904036200	≤0.5	INH, PAS
662003052840	≤0.5	INH, EMB, PZA, S, CYC, PAS, ETH
662012006551	≤0.5	INH, PAS, PZA, ETH (LNZ geno)
662101040833	≤0.5	INH, RIF, PZA, S, PAS, EMB, ETH, KAN, CAP, AMK, CYC
662105041666	≤0.5	INH, RIF, S, PZA, EMB, FQL, ETH, CYC, PAS
662108038389	≤0.5	INH, RIF, S, PAS
661906047849	≤0.5	INH, RIF, S, PZA, ETH, PAS, EMB, CYC
661907010966	≤0.5	INH, RIF, S, PAS, EMB, PZA, CYC, AMK, KAN, CAP, ETH
661908012994	≤0.5	INH, RIF, S, PZA, PAS, ETH
661911031781	≤0.5	INH, RIF, S, PAS, CYC, ETH, KAN
661911038007	≤0.5	INH, RIF, S, PAS
662004032055	≤0.5	INH, RIF, S, PAS, ETH
662001006107	≤0.5	INH, RIF, PZA, S, PAS, CYC, ETH, FQL
662001071666	≤0.5	INH, RIF, S, PAS, PZA, EMB, ETH
662001039167	≤0.5	INH, RIF, S, PAS, EMB, PZA, CYC, KAN
662008015398	≤0.5	INH, RIF, S, PAS, EMB, PZA, ETH

*INH* isoniazid, *RIF* rifampicin, *S* streptomycin, *PAS* *para*-aminosalicylic acid, *EMB* ethambutol, *PZA* pyrazinamide, *CYC* cycloserin, *ETH* ethionimide, *KAN* kanamycin, *CAP* capreomycin, *AMK* amikacin, *FQL* fluoroquinolone, *LNZ* linezolid.

Minimum Inhibitory Concentration is defined as the concentration of inhibitor where <1% of the inoculum is able to grow.

To further validate that JNJ-2901 indeed targets cytochrome bc, we measured the oxygen consumption rate using a Clark-type electrode on isolated membranes of either the wildtype *M. smegmatis* strain MC^2^155^36^, a cytochrome bc knockout strain (*M. smegmatis* Δ*qcrCAB*)^37^ or a cytochrome bd knockout strain (*M. smegmatis* Δ*cydAB*)^38^. Here, we find that JNJ-2901 inhibits oxygen consumption in both the wild-type strain and the cytochrome bd knockout strain with an IC_50_ of 89 ± 54 and 60 ± 26 nM, respectively (Fig. 1c), comparable to previous values for inhibition by Q203 on isolated membranes from *M. smegmatis* and *M. tuberculosis* (IC_50_ ~ 20 nM)^35^. In contrast, the cytochrome bc knockout strain (Δ*qcrCAB*) that depends entirely on cytochrome bd is resistant to JNJ-2901, with a modest 20% reduction of activity at 10 μM. Interestingly, while Q203 and JNJ-2901 show potent inhibition of oxygen consumption on isolated membranes from *M. smegmatis*, whole cell *M. smegmatis* is mostly resistant to inhibition by Q203 (MIC > 50 μM)^35^ while even a cytochrome bd knockout strain of *M. smegmatis* showed little sensitivity to Q203 (MIC ~ 2.5 μM)^35^, compared to the 1000-fold more sensitive *M. tuberculosis* cytochrome bd knockout strain (MIC ~ 2.0 nM, Fig. 1b).

Finally, we also measured the efficacy of JNJ-2901 in both an acute and a chronic mouse model infected with *M. tuberculosis* H37Rv-Δ*cydAB*. In the acute mouse model, treatment with either bedaquiline or JNJ-2901 has started one-week post-infection and continued for two weeks, after which lungs were harvested and plated for bacterial load enumeration (Fig. 1d), resulting in a 3.6-log reduction in bacterial load at an administered dose of 10 mg/kg of JNJ-2901. In the chronic mouse model, treatment was started four weeks post-infection and continued for eight weeks. Here, a 4.0-log reduction in bacterial load was obtained at a dose of 10 mg/kg of JNJ-2901, comparable to the 4.6-log reduction obtained by bedaquiline at 25 mg/kg (Fig. 1e). Hence, JNJ-2901 is an inhibitor of the main mycobacterial proton pump cytochrome bc and can reduce in vivo bacterial loads by four orders of magnitude.

### Generation of an *M. tuberculosis-*like cytochrome bc from *M. smegmatis*

**Fig. 2:**
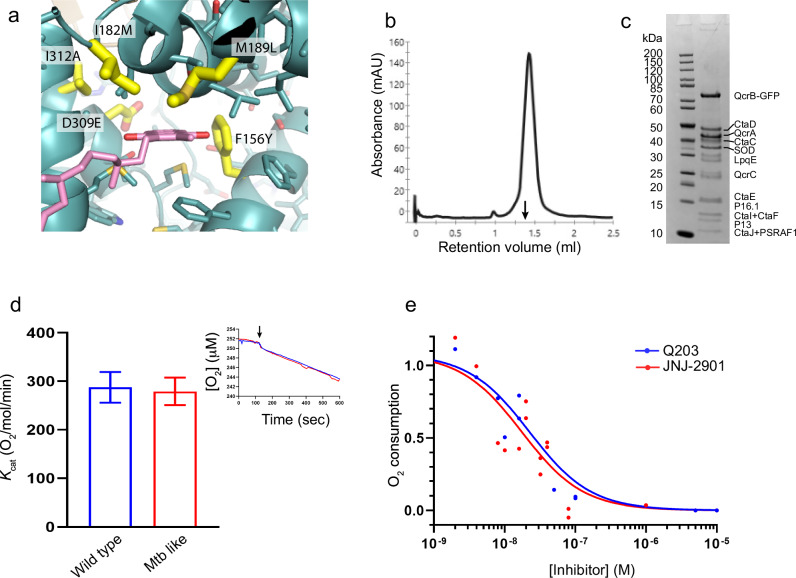
Construction and validation of an Mtb*-*like *M. smegmatis* cytochrome bc. **a** Close-up of the Qp menaquinol binding site of *M. smegmatis* cytochrome bc. Residues that differ from Mtb cytochrome bc are shown in yellow sticks. See also Supplemental Fig. 2 for sequence alignment. **b** Chromatogram showing the elution of *M. smegmatis* cytochrome bc^Mtb-like^ on a Superose 6 gelfiltration column. Vertical arrow marks the elution volume of a 670 kDa marker protein (thyroglobulin). **c** SDS–PAGE showing the purified *M. smegmatis* cytochrome bc^Mtb-like^. Subunits are indicated to the right of the gel. **d** Oxygen consumption activity by wild type (blue bar) and Mtb-like (red bar) cytochrome bc. Values were obtained from three separate experiments. Error bars indicate the standard error of the mean. Inset shows an example of oxygen consumption in the presence of cytochrome bc. Vertical arrow marks the addition of the substrate DMQH_2_. **e** Inhibitory effect of JNJ-2901 (red curve) and Q203 (blue curve) on the oxygen consumption rate of *M. smegmatis* cytochrome bc^Mtb-like^. 5 nM protein and 200 µM DMQH_2_ were used in the assay, with increasing amounts of inhibitor (5 nM–5 µM). Results were obtained from three independent experiments.

To characterize the interaction of JNJ-2901 with its target, we aimed to purify the *M. tuberculosis* cytochrome bc protein complex. However, expression of *M. tuberculosis* cytochrome bc in *M. smegmatis* results in low protein yields in our hands. In contrast, *M. smegmatis* cytochrome bc could be purified in sufficient amounts (1 mg/L of culture) and high purity. Therefore, we set out to create a version of the *M. smegmatis* cytochrome bc in which the substrate binding site is identical to that of *M. tuberculosis*. The QcrA and QcrB subunits of cytochrome bc make up the Qp menaquinone active site and share high sequence homology between *M. smegmatis* and *M. tuberculosis* (QcrA: 78% identity, QcrB: 82% identity, Supplementary Fig. 1 and 2). Only five amino acid positions in the Qp menaquinone binding site differ between *M. smegmatis* and *M. tuberculosis*: Phe156Tyr, Ile182Met, Met189Leu, Asp309Glu, and Ile312Ala (Fig. 2a, Supplementary Fig. 2). Therefore, we engineered a version of *M. smegmatis* cytochrome bc in which all five residues were mutated to the residues found in *M. tuberculosis*, which we termed cytochrome bc^Mtb-like^. Next, we purified cytochrome bc^Mtb-like^ in large amounts (0.5 mg/L of culture) and homogeneity (Fig. 2b, c) and compared the enzymatic activity of wild-type *M. smegmatis* cytochrome bc and cytochrome bc^Mtb-like^ by an oxygen consumption assay. Here, we find that the oxygen consumption activities of *M. smegmatis* wild-type cytochrome bc and cytochrome bc^Mtb-like^ are identical, indicating that the five mutations do not affect the activity of the enzyme (Fig. 2d). Finally, we measured the impact of JNJ-2901 and that of Q203 on cytochrome bc^Mtb-like^ and found that the inhibitory action of both inhibitors on cytochrome bc^Mtb-like^ are similar, with IC_50_ values of 17.4 (±4.8) and 22.4 (±8.4) nM, respectively (Fig. 2e).

### Cryo-EM structures of *M. smegmatis* cytochrome bc^Mtb-like^

**Fig. 3:**
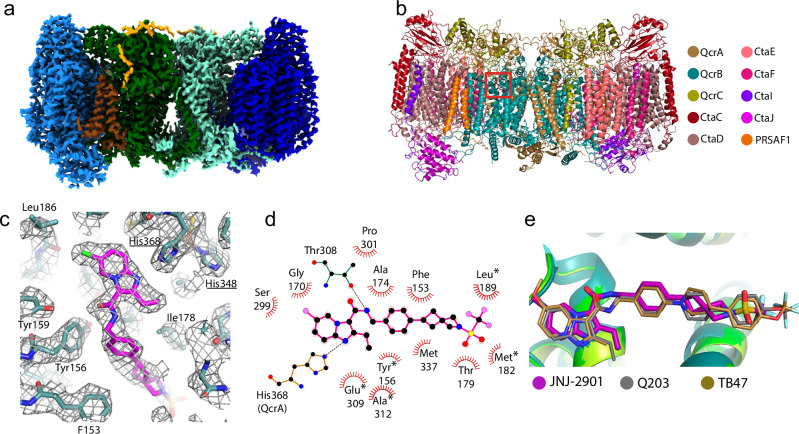
Cryo-EM structure of cytochrome bc^Mtb-like^ bound to JNJ-2901. **a** Cryo-EM map *M. smegmatis* cytochrome bc^Mtb-like^ bound to JNJ-2901. Subcomplex III (bc_1_) is shown in light and dark green, subcomplex-IV (aa_3_) is shown in light and dark blue. PSRAF1 is shown in brown, and the tail of SOD in yellow. **b** Structure of *M*. smegmatis cytochrome bc^Mtb-like^ shown in cartoon representation. The red square marks the location of the JNJ-2901 binding site in one of the two copies of subcomplex III. **c** Close-up of JNJ-2901 binding site. QcrB is shown in teal, JNJ-2901 in magenta, and cryo-EM map is shown in gray mesh. Underlined residues (His348 & His368) are from the QcrA subunit, all other residues are from the QcrB subunit. **d** Schematic representation of the interactions between cytochrome bc and JNJ-2901. Hydrophobic interactions are indicated by striated half circles and hydrogen bonds by dashed black lines. Residues mutated to make cytochrome bc^Mtb-like^ are marked by a star. **e** Superimposition of three cytochrome bc inhibitors: JNJ-2901 (magenta)_,_ Q203 (gray, PDB:7E1W) and TB47 (sand, PDB:7E1X).

To establish how JNJ-2901 interacts with cytochrome bc, we determined the cryo-EM structure of *M. smegmatis* cytochrome bc^Mtb-like^ bound to JNJ-2901 to a resolution of 3.1 Å (Fig. 3, Table 2, Supplementary Fig. 3). Overall, the structure is identical to previous structures of mycobacterial cytochrome bc^39^. It forms a ‘dimer of dimers’, containing two copies of subcomplex III (bc_1_) and two copies of subcomplex IV (aa_3_) (Fig. 3a). Subcomplex III consists of the three subunits: QcrA, QcrB, and QcrC, while subcomplex IV consists of six subunits: CtaC, CtaD, CtaE, CtaF, CtaI, and CtaF, complemented by PRSAF1 (Fig. 3b). At a low map-contour level, the cryo-EM map shows density for the Super Oxide Dismutase (Supplementary Fig. 3e), which also in other publications has been reported to be poorly defined in the cryo-EM map due to its flexibility^39^. JNJ-2901 is bound in the Qp menaquinol binding site (Fig. 3c), located in the QcrB subunit of the subcomplex III. This is the same binding site as for the other cytochrome bc inhibitors Q203 and TB47^24,25^. The density for JNJ-2901 is well defined allowing for an accurate positioning of the inhibitor. Only the end of the tail of the inhibitor shows weaker density as it is located out of the ligand binding pocket and has no interaction with the protein. The interactions with the inhibitor are mainly hydrophobic in nature (Fig. 3d) with only two hydrogen bonds between the protein and inhibitor. Almost all contacts are with the QcrB subunit, with a single contact from the QcrA subunit. The five residues that we mutated to make the *M. smegmatis* ligand binding site identical to *M. tuberculosis* all interact with JNJ-2901 (marked with a star in Fig. 3d). The cryo-EM map for these residues is well resolved and fits well with the mutated amino acids, but not the original wild type amino acids (Supplementary Fig. 4). Comparison of the position of JNJ-2901 to that of other cytochrome bc inhibitors Q203 and TB47 shows a similar configuration in which the three inhibitors overlap well (Fig. 3e) with the main difference in the tail region, which has few contacts with the protein. The high similarity in binding pose agrees with the similar IC_50_ values for JNJ-2901 and Q203 on the isolated cytochrome bc^Mtb-like^ protein (Fig. 2e).

**Table 2:** Cryo-EM data collection and data refinement data

	Cytochrome bc/JNJ-2901
*Data collection*	
Microscope	Titan Krios
Voltage (kV)	300
Magnification	×105,000
Detector—GIF	Gatan K3—Bioquantum
Data collection software	EPU
Electron exposure (e^–^/Å^2^)	50
Defocus range (μm)	0.8–2.5
Pixel size (Å)	0.836
*Data processing*	
Software	Relion 4
Number of micrographs	4522
Final number of particles	42,116
Symmetry imposed	C1
Map resolution (Å)	3.1
FSC threshold	0.143
*Model refinement*	
Software	Phenix 1.18.2
Map correlation coefficient	0.83
Model composition	
Number of chains	22
Non-hydrogen atoms	43,307
Protein residues	5206
Ligands	59
*Validation*	
R.M.S. deviations	
Bond lengths (Å)	0.004
Bond angles (°)	0.814
Ramachandran plot	
Favored (%)	96.6
Allowed (%)	3.4
Disallowed (%)	0
Rotamer outliers (%)	0
*Data availability*	
EMDB entry	EMD-51689
PDB entry	9GY6

### Analysis of JNJ-2901 resistance-inducing mutations

**Fig. 4:**
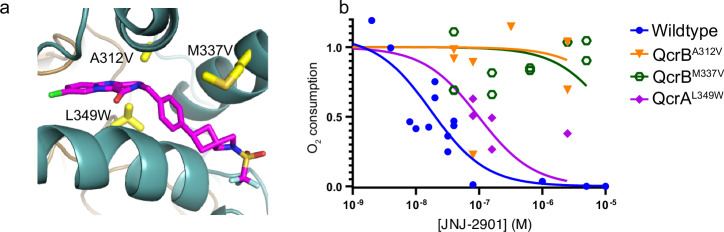
JNJ-2901 resistance mutatations. **a** Close-up of the JNJ-2901 binding pocket with JNJ-2901 shown in magenta and mutated residues shown in yellow sticks. **b** Sensitivity of wild type and JNJ-2901-resistant mutants cytochrome bc protein as measured in an oxygen consumption assay. Reaction mixtures contained 30 nM protein and 200 µM DMQH_2_, with increasing amounts (5 nM–5 µM) of inhibitor. Results are shown of four independent experiments per protein variant.

One of the main challenges in the treatment of tuberculosis is the frequent occurrence of resistance against antibiotics^1,40–42^. Also, for the cytochrome bc inhibitors Q203 and TB47, resistance-inducing mutations have been reported^17,34,43–53^ Therefore, we wanted to determine if *M. tuberculosis* can also develop resistance against JNJ-2901. For this, *M. tuberculosis* H37Rv-Δ*cydAB* was exposed to a high concentration of JNJ-2901 (100 × MIC) and grown for four weeks. After incubation, single colonies were picked and submitted for genome sequencing, revealing twelve mutations that correspond to three amino acid positions located in the ligand binding pocket of cytochrome bc (Table 3, Fig. 4a). Two of these are in the QcrB subunit, QcrB^A317V^ (10×) and QcrB^M342T^ (1×), and one in the QcrA subunit, QcrA^L356W^ (1×). Changes in these amino acid positions have also been observed in laboratory-derived mutations that drive resistance against Q203 and TB47^17,23,47,53,54^ (Supplementary Table 1). To further characterize the JNJ-2901 resistance-inducing mutations, we created three variants of *M. smegmatis* cytochrome bc^Mtb-like^: QcrB^A312V^, QcrB^M337V^, or QcrA^L349W^ (Table 3, Fig. 4a) and expressed and purified the mutant proteins. Next, we compared the inhibitory effect of JNJ-2901 on the activity of these mutant proteins. We find that all three mutants show a reduced sensitivity to JNJ-2901 (Fig. 4b), with the two mutants in the QcrB subunit showing more than a 700-fold increase in IC_50_ when compared to the wild-type protein (wild-type: 19 nM, QcrB^A312V^: 23.8 µM, QcrB^M337V^: 12.9 µM), while the QcrA^L349W^ mutation showing a modest 5-fold increase in IC_50_ compared to wild type protein (QcrA^L349W^: 98 nM). Both QcrB^A312^ and QcrB^M337^ are part of the hydrophobic network that interacts with JNJ-2901 (Fig. 3d), while QcrA^L349^ is located further away (at 4.1 Å) and is positioned next to a cavity that could accommodate the larger sidechain of a tryptophane. This could explain the lesser impact of this mutation on the IC_50_ of JNJ-2901. These mutant proteins will further serve as tools for the screening of novel inhibitors targeting the resistant enzyme.

**Table 3:** JNJ-2901 resistance mutations

Subunit	Mutation in Mtb	*M. smegmatis* residue	Mutant protein
QcrB	A317V	A312	A312V
QcrB	M342T	M337	M337V
QcrA	L356W	L349	L349W

List of mutations in Mtb H37Rv-Δ*cydAB* that give rise to resistance against JNJ-2901.

## Discussion

The widespread prevalence of tuberculosis, combined with an increase in antibiotic-resistant cases, is a major threat to the world’s health. To combat antibiotic resistance, new drugs that inhibit novel targets are needed. The bacterial proton pump cytochrome bc is at the heart of the bacterial respiratory chain, and it is critical for the generation of ATP in bacteria. Novel inhibitors that act on cytochrome bc such as Telacebec (Q203), have passed phase 1 clinical trials and showed promising results in phase 2a^20,55^, while TB47 is currently in pre-clinical studies. To ensure that a cytochrome bc inhibitor will reach the treatment stage it is important to include new compounds in the drug development pipeline. In this work, we show that JNJ-2901, a novel analog of Q203 and TB47, is a potent growth inhibitor of clinical strains of *M. tuberculosis* in vitro, with a MIC of less than 1 nM. In addition, JNJ-2901 is effective in both an acute and chronic mouse model infected with *M. tuberculosis* H37Rv-Δ*cydAB*, resulting in a 4-log reduction of bacterial load in the lungs, similar to the reduction observed for the widely used bedaquiline.

The cryo-EM structure of cytochrome bc bound to JNJ-2901 shows a canonical binding mode like other inhibitors with an extensive network of hydrophobic interaction. We furthermore show that three resistance-inducing mutations are positioned in the inhibitor binding pocket and that two of these mutations increase the IC50 by >700-fold. It should be noted that these mutations were generated in a laboratory setting and do not necessarily reflect resistance mutations that may occur in a clinical setting. Moreover, current treatment regimens for drug-susceptible and drug-resistant *M. tuberculosis* include multiple antibiotics to reduce the chances of developing drug resistance. Likewise, we envision that JNJ-2901 should also be used in combination with other antibiotics. Therefore, in a parallel publication, we show that JNJ-2901 is a good partner for the BPaL regimen that is currently used for drug-resistant TB (Aguilar-Perez, submitted, 10.21203/rs.3.rs-5331796/v1) and could be used as a replacement for moxifloxacin in fluoroquinolone-resistant TB. Taken together, our work shows that JNJ-2901 is a promising novel inhibitor of *M. tuberculosis* cytochrome bc, with the potential of becoming an asset in the ongoing battle against TB. JNJ-2901 should also be further studied as a therapeutic option for Buruli ulcer^56^, a neglected tropical disease caused by *Mycobacterium ulcerans*, and Leprosy^57^ caused by *Mycobacterium leprae*. Both mycobacteria lack the gene encoding cytochrome bd in their genome, indicating that they rely completely on cytochrome bc for proton pump activity^58,59^. This is validated by cytochrome bc inhibitors showing promising results in this direction^46,60,61^.

## Methods

### Chemicals and DNA primers

All chemicals were purchased from Sigma-Aldrich unless stated otherwise. DNA oligos were purchased from IDT-DNA.

### Bacterial strains and growth conditions

The *M. tuberculosis* H37Rv strain was kindly provided by Roland Brosch (Institut Pasteur, France). To prepare frozen stocks, H37Rv was grown in Middlebrook 7H9 (Becton-Dickinson) culture medium supplemented with 10% oleic acid–albumin–dextrose–catalase (OADC) complex (Becton-Dickinson) and 0.05% Tween 80 (Sigma-Aldrich). Upon reaching the stationary phase, the H37Rv culture was harvested in glycerol (15%) (Becton-Dickinson) containing Middlebrook 7H9 medium (supplemented with 10% OADC and 0.05% Tween 80) and frozen at –80 °C.

### Minimal inhibitory concentration determination on clinical isolates of *M. tuberculosis*

18 *M. tuberculosis* clinical strains harboring various drug susceptibility profiles were selected from the French NRC of mycobacteria collection (including 13 MDR-TB strains). Drug susceptibility profile was determined by using the BACTEC™ MGIT 960 system for the first-line drug and the reference proportion method in Löwenstein-Jensen for second-line drug.

The agar dilution method was performed on 7H11 supplemented with 10% Middlebrook OADC (oleic acid, albumin, dextrose, and catalase) (BD). Two-fold dilutions of the compounds were added to obtain the final concentrations. A 1/100 dilution of a McFarland 1.0 turbidity standard suspension was inoculated with a Steers replicator delivering approximately 10^4^ CFU per spot. Plates were incubated at 37 °C and MICs were determined after 4 weeks of incubation. The MIC was defined as the lowest concentration of antibiotic resulting in complete inhibition of growth or in the growth of fewer than 10 colonies (<1% of the inoculum).

### Minimal inhibitory concentration determination on lab strain *M. tuberculosis* H37Rv Δ*cydAB*

Compounds screened in dose-response were tested in 4-fold dilutions from 5 mM to 0.005 µM in black, clear bottom, 384-well microtiter plates (Greiner). Using an Echo liquid handler (Labcyte Inc., Sunnyvale, CA, USA) a low volume dilution range in 100% DMSO was dispensed in the plates (150 nl/well). Reference plates were included as well as positive and negative control wells in each plate. *M. tuberculosis* H37Rv Δ*cydAB* (1 × 10^5^ CFU/m) diluted in Middlebrook 7H9 medium supplemented with 10% OADC, 0.5% glycerol, and 0.05% Tween 80 was added to the compound plates (30 µL). Plates were stacked in iron racks and incubated for 5 days at 37 °C. Prior absorbance measurements (OD_620_) using an Envision multimode plate reader (Perkin Elmer) the pates were shaken (4 min, 1000 rpm). MIC_50_ values were determined as the drug concentration inhibiting 50% of the growth observed in the control wells.

### Generation of JNJ-2901 resistant strains in *M. tuberculosis* H37Rv Δ*cydAB*

To isolate resistant colonies, agar plates containing 100 × MIC90 of JNJ-2901 were inoculated with *M. tuberculosis* H37Rv Δ*cydAB* (5 × 10^8^ CFU) to select resistant colonies. Individual colonies were re-plated in the presence of the compound for ~3 weeks to confirm resistance. Genomic DNA was isolated from resistant colonies using the Quick-DNA fungal and bacterial miniprep kit (Zymo Research). WGS libraries were prepared using the Nextera XT DNA Library Preparation Kit (Illumina). Sequencing was performed on an Illumina NextSeq 550 platform, generating paired-end 75 bp reads and targeting 5 M sequences per sample. The trimmed reads were mapped to the reference genome of *M*. *tuberculosis* H37Rv (GenBank accession number NC_000962.3) and to the *M. tuberculosis* H37Rv *ΔcydAB* control. Variants were identified using the CLC Genomics Workbench v21.0.5 (Qiagen) variant caller, with a minimum count of 2, minimum coverage of 10, and a minimum frequency of 10%. Variants present in all samples and wild-type samples were filtered out. Only variants present in >75% of the reads were considered to minimize noise.

### Animal Ethics statement

All the in vivo studies were performed at Johnson & Johnson Innovative Medicines in Beerse, in a certified BSL3 facility in agreement with European Directive (2010/63/EU) and national directives for the protection of animals for experimental purposes. All procedures were approved by the Ethics Committee of Johnson & Johnson Innovative Medicines, located in Beerse, Belgium, which has been accredited by AAALAC since 2004 under unit number 001131 (https://www.aaalac.org/).

### Mice

Six to eight weeks old female Balb/cBy mice were purchased from Charles-River (France) or Janvier (France). Mice were allocated in the BLS3 facility in individually ventilated cages in HEPA-filtered racks and rested for at least 5 days to allow acclimatization. Access to water and food was ad libitum. General anesthesia was practiced to infect mice intranasally and for unconscious euthanasia followed by cervical dislocation. Groups of 6 mice were placed in the anesthesia box (integrated gas anesthesia system of the Techniplast Aria Tech60 GAS biosafety cabinet using GE HealthCare Tec 850 vaporizer). Anesthesia was performed using inhalation of isoflurane (Alivira Isoflutek 1000) at 5% for induction and 2.5–3% for maintenance.

### Short acute model

The acute mouse model infections were performed as described in refs. ^62^^,^^63^. Mice were infected intranasally with 200 cfu per mouse. To verify the infection level a subgroup of 6 mice were sacrificed one day after the infection. Mice were infected for a week when treatment started, mice were treated daily during 12 consecutive days, mice were euthanized 3 days after the last dose to prevent carry over effect. To control the evolution of the infection, a group of mice was euthanized 7 days post-infection, when treatment starts; and 21 days post-infection, when treatment has ended. Negative control mice remained untreated. Mice were dosed by oral gavage (100 µL, drencher with rounded end straight, 0.9 mm × 25 mm, Socorex Swiss) except for the long-acting formulation that was injected subcutaneously in the upper back (100 µL) using a needle (26Gx13mm, BD Microlance^TM^). At sacrifice, whole lungs are aseptically collected in Gentlemacs tubes (M tubes with strainer, Miltenyi Biotec) containing 2 ml of PBS and homogenized using the RNA_01_01 settings of GentleMACS^TM^ Octo Dissociator (Miltenyi Biotec). Lung homogenate was diluted in PBS and plated in 7H10 charcoal agar plates containing antibiotics (amphotericin: 100 µg/ml, Polymyxin B: 25 µg/ml, Carbenecillin: 50 µg/ml, Trimethoprim: 20 µg/ml 0.5 mg/ml). Plates were incubated at 37 °C during 3–5 weeks. After that, CFU counts were recorded, and data was expressed in the mean log CFU/lung for each group. Statistical analysis was done by one-way ANOVA with Sidak’s test for multiple comparisons in GraphPad Prism.

### Chronic model

The chronic mouse model infections were performed as described in ref. ^64^. Mice were infected intranasally with 200 cfu per mouse. To verify the infection level a subgroup of 6 mice were sacrificed one day after the infection. Mice were infected for a month before treatment started; treatment lasted for 8 weeks. Six mice were sacrificed at the start of treatment as pretreatment control. Negative control mice remained untreated. Mice were dosed by oral gavage (100 µL, drencher with rounded end straight, 0.9 mm × 25 mm, Socorex Swiss) except for the long-acting formulation that was injected subcutaneously in the upper back (100 µL) using a needle (26Gx13mm, BD Microlance^TM^). At sacrifice, whole lungs are aseptically collected in Gentlemacs tubes (M tubes with strainer, Miltenyi Biotec) containing 2 ml of PBS and homogenized using the RNA_01_01 settings of GentleMACS^TM^ Octo Dissociator (Miltenyi Biotec). Lung homogenate was diluted in PBS and plated in 7H10 charcoal agar plates containing antibiotics (amphotericin: 100 µg/ml, Polymyxin B: 25 µg/ml, Carbenecillin: 50 µg/ml, Trimethoprim: 20 µg/ml 0.5 mg/ml). Plates were incubated at 37 °C during 3–5 weeks. After that, CFU counts were recorded, and data were expressed in the mean log CFU/ lung for each group. Statistical analysis was done by one-way ANOVA with Sidak’s test for multiple comparisons in GraphPad Prism.

### Construction of a cytochrome bc expression vector

The *M. smegmatis qcrCAB* operon that encodes for complex III of cytochrome bc was cloned into an acetamide inducible plasmid pACE-GFP-His^65^ to create the expression plasmid pACE^qcrCAB-Ms^ for purification of *M. smegmatis* cytochrome bc. To make the *M. smegmatis* Qp menaquinone substrate binding site identical to that of *M. tuberculosis* cytochrome bc, five mutations (F156Y, I182M, M189L, D309E, and I112A) were introduced in the qcrB substrate binding site. This resulted in the creation of pACE^qcrCABMtb-like^, which was used for the expression and purification of *M. tuberculosis*-like *M. smegmatis* cytochrome bc (cytochrome bc^Mtb-like^) protein. JNJ-2901-resistant variants of *M. smegmatis* cytochrome bc^Mtb-like^: QcrB^T308A^, QcrB^A312V^, QcrB^M337V^, and QcrA^L349W^ were created through site-directed mutagenesis. All the primers used for cloning are listed in Supplementary Table 2.

### Protein expression and purification

Proteins were expressed in *M. smegmatis* mc^2^ 155^36^ in 1xYT media supplemented with 100 mM phosphate, 25 mM sulfate, 50 mM ammonium, 100 mM sodium, 50 mM potassium, 0.5% glycerol, 0.05% glucose, 0.2% alpha-lactose, 0.05% tween-80, and 50 µg/ml of hygromycin. A cell culture at OD_600_ ~ 1.5 was induced with 0.04% acetamide and grown for 6 h at 37 °C, harvested and flash frozen in liquid nitrogen, and stored at −80 °C.

Cell pellets were resuspended in lysis buffer (25 mM HEPES, pH 7.5, 25 mM imidazole pH 7.5, and 100 mM NaCl) and lysed by passing through an Emulsifex-C5 cell disruptor (Avestin, Inc.). The lysate was spun at 6000 × *g* to remove the unlysed cells, and then the supernatant was at 257,000 × *g* for 1 h. The isolated membrane was resuspended in solubilization buffer (25 mM HEPES pH 7.5, 25 mM imidazole pH 7.5 and 100 mM NaCl). Then 1% DDM (n-Dodecyl-ß-d-maltoside) detergent was added and incubated on a rocking platform at 4 °C for 1 h, followed by a centrifugation step at 17,000 × *g*. The supernatant was loaded onto a Ni-NTA column (Cytiva) and pre-equilibrated in a solubilization buffer with 0.05% DDM. Cytochrome bc was eluted from the column with a gradient of 25–500 mM imidazole in elution buffer (25 mM HEPES pH 7.5, 100 mM NaCl, 0.05% DDM). The protein was concentrated in a 100 kDa centrifugal filter device (Amicon Ultra) and injected onto a pre-equilibrated Superose 6 Increase size exclusion column (GE Healthcare) and run with gel filtration buffer (50 mM HEPES pH 7.5, 100 mM NaCl, and 0.02% DDM). The protein was concentrated to 5 µM, flash frozen in liquid nitrogen, and stored at −80 °C.

### Oxygen consumption assay on membrane vesicles

Membrane fractions were prepared from *M. smegmatis strains* mc^2^ 155 wild type, an *M. smegmatis* cytochrome bd knockout strain (*cydA*::*hyg*), and an *M. smegmatis* cytochrome bc knockout strain (*qcrABC*::*hyg*). Oxygen consumption assays were performed using membrane vesicles with a total of 30 µg of protein and 200 µM NADH in 50 mM HEPES pH 7.5, 100 mM NaCl in a final volume of 600 µl at 37 °C. For the determination of IC_50_ values, increasing amounts of inhibitor were added from 5 nM to 5 µM.

### Oxygen consumption assay on purified protein

Cytochrome bc enzymatic activity was measured in an Oxytherm+ instrument (Hensatech Instrument Ltd.) as described in ref. ^24^ with a few modifications. The menaquinol mimic 2,3-dimethyl-[1,4]naphthohydroquinone (DMW) (Enamine) was reduced by mixing a 1:10 molar ratio of DMW and DTT for 10 mins at 37 °C to form DMWH_2_. To start the assay 5 nM of the enzyme, 50 µM of DMWH_2_, and 50 nM bovine SOD were mixed in 50 mM HEPES pH 7.5, 100 mM NaCl in a final volume of 600 µl and oxygen consumption was measured for 2 mins at room temperature. For the determination of IC_50_ values, increasing amounts of inhibitor were added from 5 nM to 5 µM.

### Cryo-EM sample preparation and imaging

Quantifoil grids (R 2/1, 300 mesh) were glow-discharged using the EMITECH K950 instrument (Quorum) with 20 mA current in 0.2 m Torr vacuum for 45 s. 3 µl of 5 µM purified cytochrome bc^Mtb-like^ complex was mixed with JNJ-2901 in a 1:10 molar ratio and applied on the glow discharged grid. The grid was blotted in 85% humidity at 6 °C for 1 s and plunge frozen in liquid ethane using a GP2 instrument (Leica). Cryo-EM data were collected at The Netherlands Center for Electron Nanoscopy (NeCEN) on a Titan Krios (FEI) electron microscope operating at 300 kV with a K3 direct electron detector equipped with a BioQuantum energy filter (Gatan) set to 20 eV. Images were recorded at ×105.000 magnification, 0.836 Å pixel size, and a total dose of 50 e^−^/Å^2^, using defocus values from 0.8 to 2.5 µm. Image recording was done in counting mode in EPU software (Thermo Fisher Scientific). The parameters used for image collection are listed in Table 1.

### Cryo-EM image processing

Cryo-EM images were processed in Relion 4.0^66^. Images were motion-corrected using Motioncor2^67^ and defocus was estimated using Gctf^68^. Laplacian-of-Gaussian-based (LoG) autopicking was performed on a subset of micrographs and picked particles were two-dimensionally (2D) classified. Selected classes from the 2D classification were used as references to autopick particles from the full dataset, resulting in 678,771 particles from 4522 micrographs. After three rounds of 2D classification, 192,000 particles derived from classes with different orientations were selected for 3D classification. One of the 3D classes with 42,000 particles was further 3D refined. Defocus values were further refined using CTF Refinement in Relion, followed by Bayesian polishing. Another round of 3D autorefinement was performed on these polished particles. The map was post-processed to correct for the modulation transfer function of the detector and sharpened by applying a negative *B* factor, as determined automatically by Relion. A soft mask was applied during postprocessing to generate the FSC curve. A starting model of *M. smegmatis* cytochrome bc (PDB code: 6ADQ^22^, was fitted to the cryo-EM map using Coot^69^ and refined in Phenix^70^. An atomic model for JNJ-2901 was generated using the ligand builder in Coot, and a geometry restraint file was generated in PRODRG^71^. Figures were prepared using Pymol^72^, ChimeraX^73^, and LigPlot^+^^74^. Details on data processing, model refinement, and validation are given in Table 1 and Supplementary Fig. 3.

## Supplementary information

Supplementary information

## Data Availability

The datasets used in this study are available from the corresponding author on request. The cryo-EM map has been submitted to the EMDB under access code: EMD-51689. The molecular model has been submitted to the wwwPDB under access code: 9GY6.
