# Supplementary material for: Structural and mechanistic study of a novel inhibitor analogue of M. tuberculosis cytochrome bc1:aa3
Source: NPJ Drug Discov. 2025 Apr 2;2:6. doi: 10.1038/s44386-025-00008-3 (PMC11964921; doi:10.1038/s44386-025-00008-3)
Supplement: Supplementary file 1 — Supplementary information [file 44386_2025_8_MOESM1_ESM.pdf]

Supplementary Information

Structural and mechanistic study of a novel inhibitor analogue of *M. tuberculosis* cytochrome $bc_1:aa_3$

Amit Kumar Verma[1,6], Robbert Q. Kim[1], Dirk Lamprecht[2], Clara Aguilar-Pérez[2], Sarah Wong[3], Nicolas Veziris[3], Alexandra Aubry[3], José Manuel Bartolomé-Nebreda[4], Rodrigo J. Carbajo[5], Jennefer Wetzel[2], and Meindert H. Lamers[1,6]

[1] Department of Cell and Chemical Biology, Leiden University Medical Center, Einthovenweg 20, 2333 ZC, Leiden, The Netherlands. [2] Janssen Pharmaceutica, Global Public Health, Turnhoutseweg 30, 2340 Beerse, Belgium, [3] Sorbonne Université, INSERM, Centre d'Immunologie et des Maladies Infectieuses, U1135, AP-HP. Sorbonne-Université, Fédération de Bactériologie, Centre National de Référence des Mycobactéries et de la Résistance des Mycobactéries aux Antituberculeux, Paris, France, [4] Global Discovery Chemistry, Janssen-Cilag, S.A., a Johnson & Johnson Innovative Medicine company, c/ Jarama, 75A, 45007 Toledo, Spain, [5] In Silico Discovery, Janssen-Cilag, S.A., a Johnson & Johnson Innovative Medicine company, c/ Jarama, 75A, 45007 Toledo, Spain.

[6] Correspondence to Meindert H. Lamers (m.h.lamers@lumc.nl) or Amit K. Verma (a.k.verma1@lumc.nl)

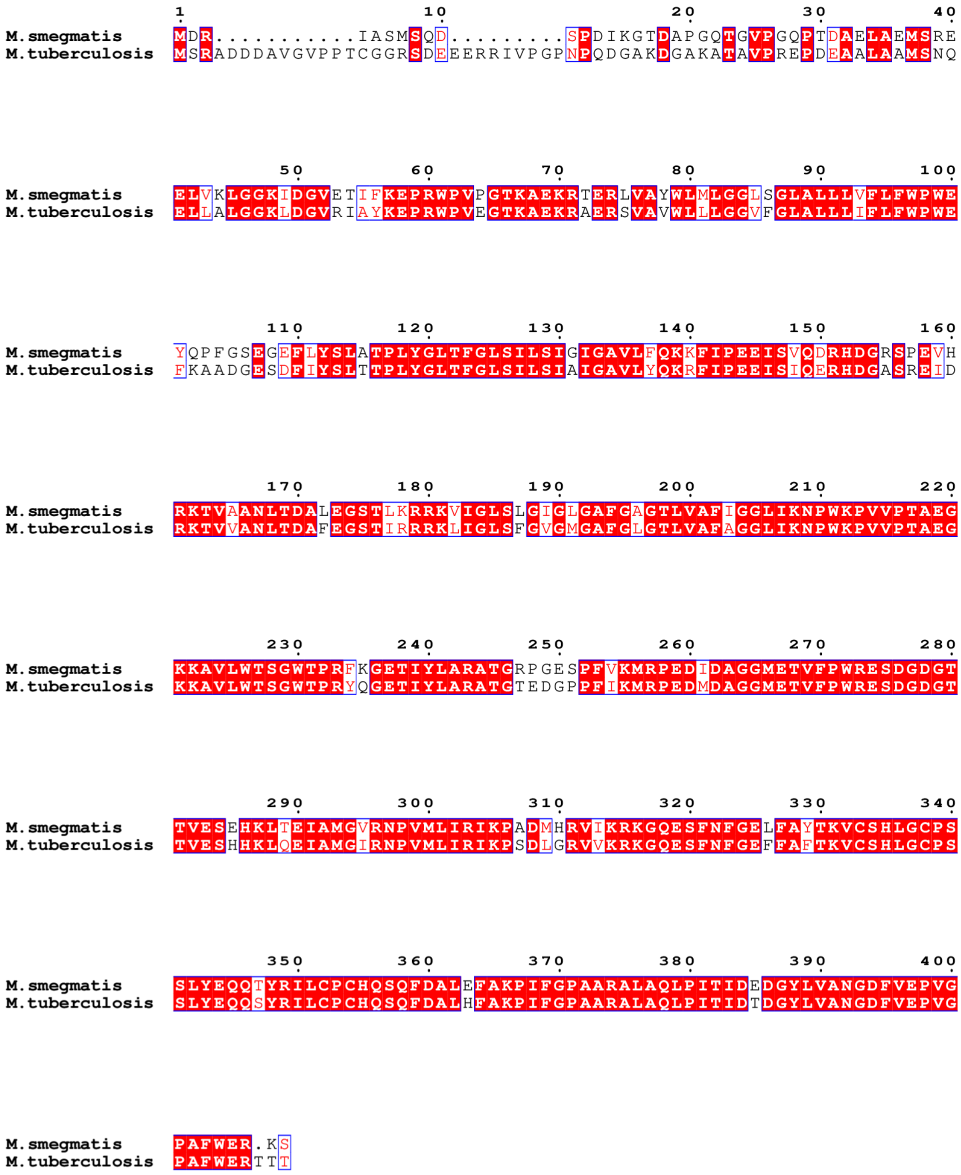

Supplementary Figure 1: Sequence conservation of cytochrome bc QcrA subunit. Top sequence: *M. smegmatis* QcrA, bottom sequence: *M. tuberculosis* QcrA.

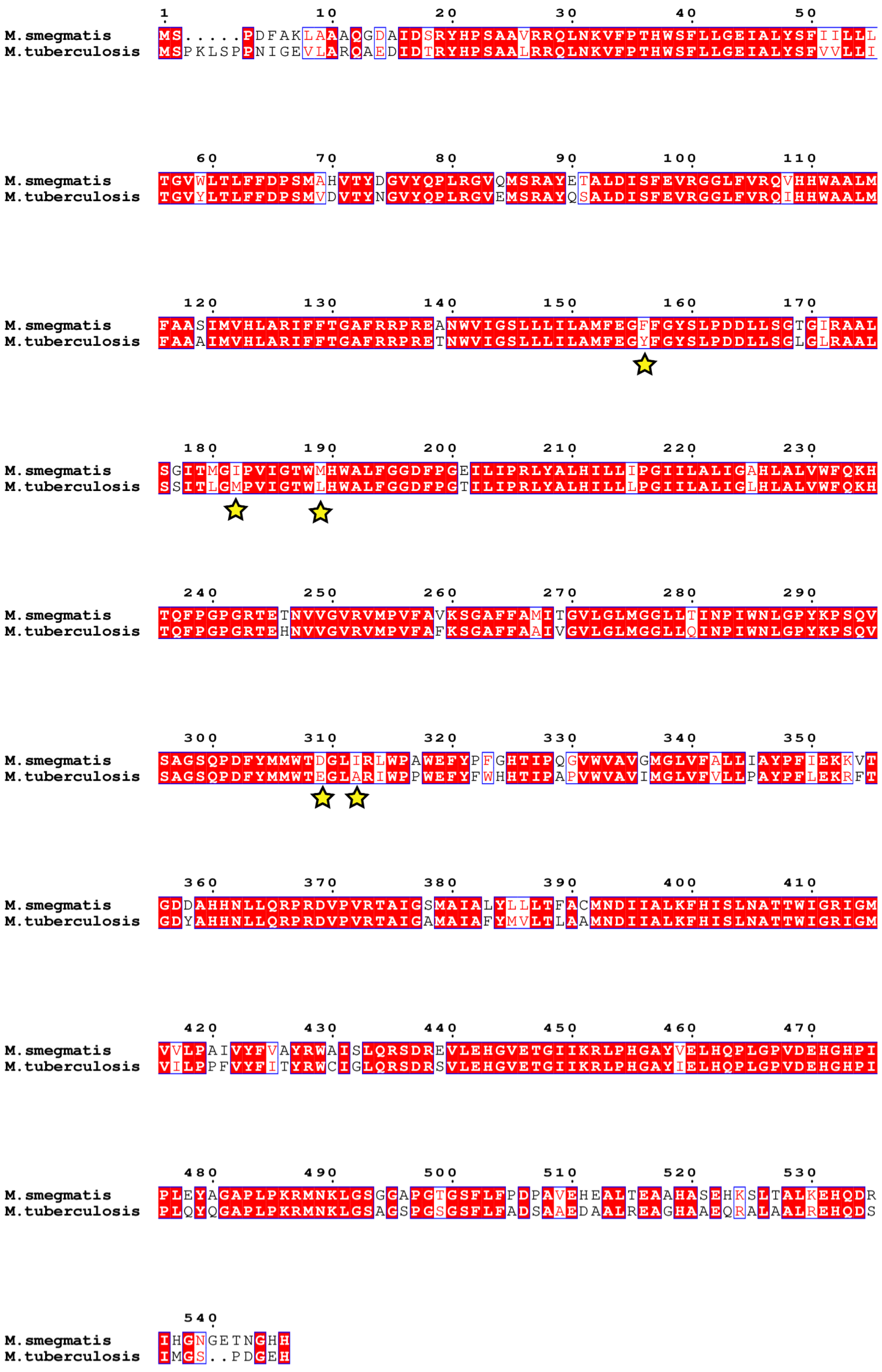

Supplementary Figure 2: Sequence conservation of cytochrome bc QcrB subunit. Top sequence: *M. smegmatis* QcrB, bottom sequence: *M. tuberculosis* QcrB. The yellow stars under the sequence mark the five residues in the Qp menaquinol binding site that differ between *M. smegmatis* and *M. tuberculosis*.

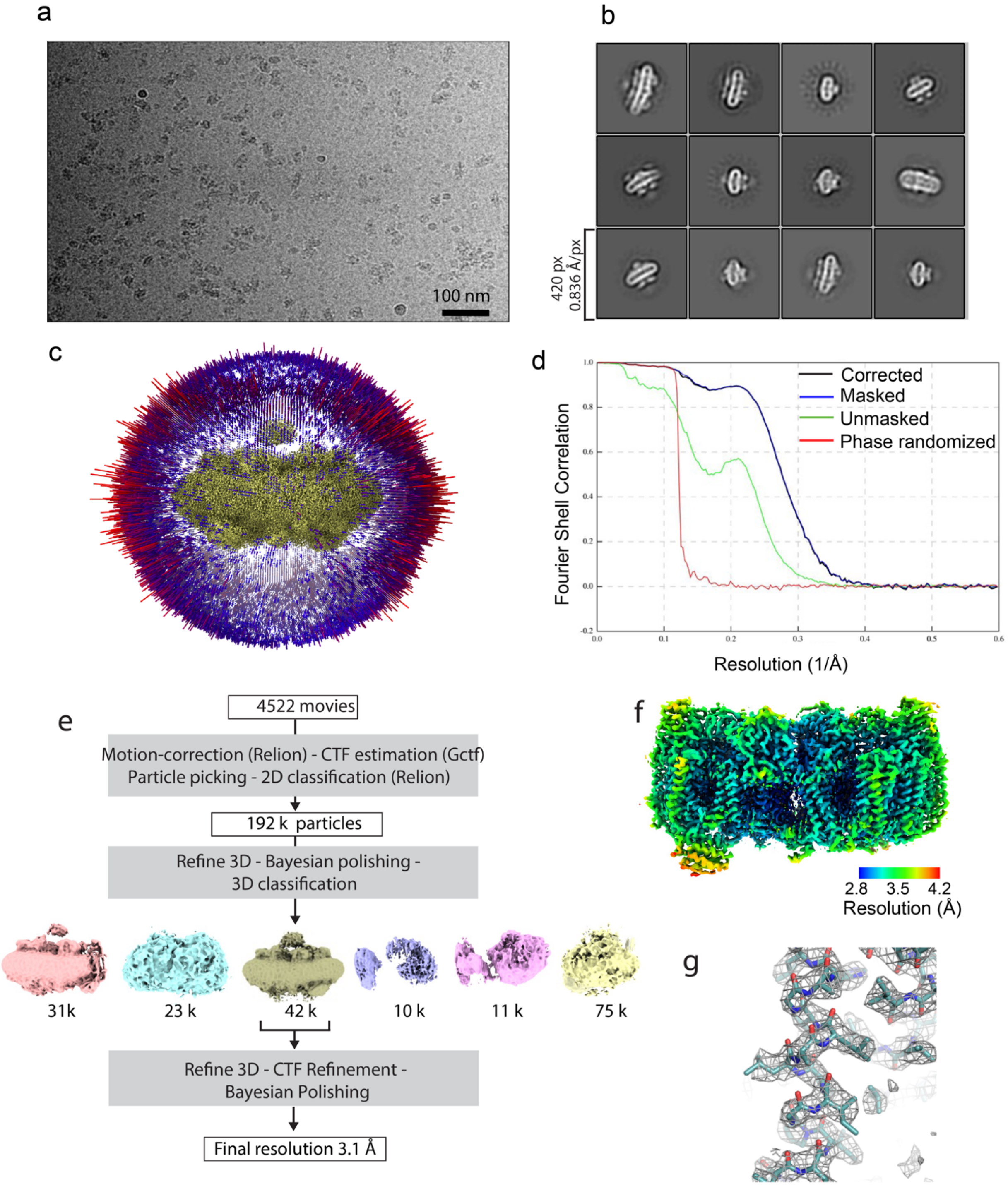

Supplementary Figure 3. Structure determination of *M. smegmatis* cytochrome bc$^{Mtb\text{-}like}$. (**a**) cryo-EM micrograph of cytochrome bc. (**b**) Representative 2D classes. (**c**) Orientational distribution of final set of particles (**d**) Fourier Shell Correlation between half-maps at sequential stages of the refinement process. (**e**) Schematic representation of data processing workflow. (**f**) Local resolution map. (**g**) Representative view of the cryo-EM map in grey mesh and fitted model in teal sticks.

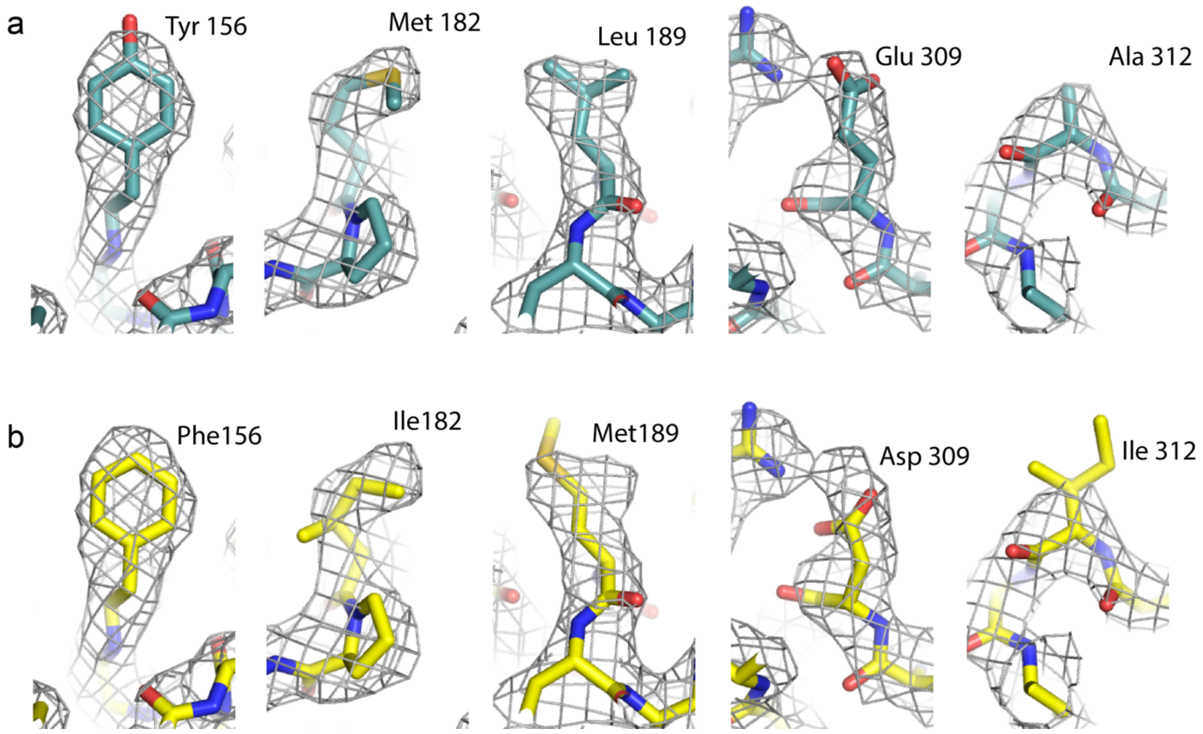

Supplementary Figure 4. Comparison of the fit of the mutated vs original residues in to the cryo-EM map. (a) The five mutated resides (in teal sticks) fitted into the cryo-EM map (in grey mesh), (b) The five the original, wild type residues (in yellow sticks) fitted into the cryo-EM map. For all mutations, the mutated residues show a better fit into the map than the original wild type residues.

Subunit	Mutation	Inhibitor	Reference
QcrB	G175S	CWHM-728	(Harrison-2019)
QcrB	G175S	Quinazoline	(Lupien-2020)
QcrB	L176P	lanzaprazole	(Rybniker-2015)
QcrB	A178T	CWHM-728	(Harrison-2019)
QcrB	A178V	CWHM-728	(Harrison-2019)
QcrB	A179P	PABs	(Chandrasekera-2015)
QcrB	S182P	IPA	(Arora-2014)
QcrB	S182P	AX-35	(Foo-2018)
QcrB	S182P	Q203	(Moraski-2016)
QcrB	W312G	IPA	(Arora-2014)
QcrB	W312G	Q203	(Moraski-2016)
QcrB	W312G	PABs	(Chandrasekera-2015)
QcrB	W312C	PABs	(Chandrasekera-2015)
QcrB	T313I	Q203	(Pethe-2013)
QcrB	T313I	TB47	(Waller-2023)
QcrB	T313I	MOT	(Cleghorn-2018)
QcrB	T313A	Q203	(Pethe-2013)
QcrB	T313A	TB47	(Waller-2023)
QcrB	G315S	CWHM-728	(Harrison-2019)
QcrB	A317V	JNJ-2901	This work
QcrB	A317V	IPA	(Arora-2014)
QcrB	A317V	Q203	(Moraski-2016)
QcrB	A317T	IPA	(Arora-2014)
QcrB	A317T	Q203	(Moraski-2016)
QcrB	A317I	P-P-D[1]	(van der Westhuyzen-2015)
QcrB	V338G	CWHM-728	(Harrison-2019)
QcrB	M342T	JNJ-2901	This work
QcrB	M342T	IPA	(Arora-2014)
QcrB	M342T	Q203	(Moraski-2016)
QcrB	M342T	Q-Y-A[2]	(Phumerian 2016)
QcrB	M342T	MOT	(Cleghorn-2018)
QcrB	M342T	PABs	(Chandrasekera-2015)
QcrB	M342V	AX-35	(Foo-2018)
QcrB	M342I	IPA	(Arora-2014)
QcrB	M342I	Q203	(Moraski-2016)
QcrB	M342I	TB47	(Waller 2023)
QcrB	M342I	AX-36	(Foo-2019)
QcrB	M342I	Q-Y-A[2]	(Phumerian 2016)
QcrB	A396T	IPA	(Arora-2014)
QcrB	A396T	Q203	(Moraski-2016)
QcrB	A296T	Q-Y-A[2]	(Phumerian 2016)
QcrA	L356W	JNJ-2901	This work
QcrA	L356W	Lanzoprazol	(Rybniker-2015)
QcrA	L356W	Q203	(Gries 2023)
QcrA	L356V	Quinazoline	(Lupien-2020)

Supplementary Table 1. Cytochrome bc resistance mutations. Reported mutations in Mtb cytochrome bc that give rise to resistance against various inhibitors. [1] P-P-D: Pyrrolo[3,4-c]pyridine-1,3(2H)-dione, [2] Q-Y-A: 2-(quinolin-4-yloxy)acetamide.

Name	Sequence (5'-3')	Role
p1	tgcctgcaggtcgactctagaggtggatcgcgctgttcgccacc	Amplification of *qcrCAB* operon
p2	gctcggtacccggggatctcagtgatgaccgttggtctccccgttgc	Amplification of *qcrCAB* operon
p3	ggtcaggagagcttcaacttcggtgagctTttcg	Delete Sap1 restricting site on qcrA
p4	cgaagttgaagctctcctgacccttgc	Delete Sap1 restricting site on qcrA
p5	atatatGCTCTTCtAGTgatcgTgctgttcgccaccatctacttcg	Subcloning *qcrABC* into pINIT
p6	tatataGCTCTTCaTGCgtgatgaccgttggtctccccgttgcc	Subcloning *qcrABC* into pINIT
p7	tctacaccgcgatgctgacg	Sequencing of *qcrCAB* operon
p8	gcctgatcaagaacccgtgg	Sequencing of *qcrCAB* operon
p9	tgtacagcttcatcatcctgc	Sequencing of *qcrCAB* operon
p10	gatgttcgagggctacttcggttactcg	Create $QcrB^{F156Y}$ mutation
p11	cgagtaaccgaagtagccctcgaacatcg	Create $QcrB^{F156Y}$ mutation
p12	atgggtatGcccgtcatcggcacctggCtgcactgggc	Create $QcrB^{I182M}$ and $QcrB^{M189L}$
p13	agtgcagccaggtgccgatgacgggcatacccatcg	Create $QcrB^{I182M}$ and $QcrB^{M189L}$
p14	atgatgtggaccgaGggtctggcccgtctgtggccg	Create $QcrB^{D309E}$ and $QcrB^{I112A}$
p15	ccacagacgggccagaccctcggtccacatcatgtagaagtccgg	Create $QcrB^{D309E}$ and $QcrB^{I112A}$
p16	accgaGggtctgGTccgtctgtgg	Create resistant mutation $QcrB^{A312V}$
p17	ccacagacggACcagaccCtcggtcc	Create resistant mutation $QcrB^{A312V}$
p18	gtcgcggtgggcGtgggcctggtgttcg	Create resistant mutation $QcrB^{M337V}$
p19	caggcccaCgcccaccgcgacccacacg	Create resistant mutation $QcrB^{M337V}$
p20	ctgctcgcacTGgggctgcccgtcctcg	Create resistant mutation $QcrA^{L349W}$
p21	gacgggcagcccCAgtgcgagcagaccttgg	Create resistant mutation $QcrA^{L349W}$
p22	gcgttcttcgcgatgatcacc	Verify $QcrB^{A312V}$ & $QcrB^{M337V}$
p23	gcctgatcaagaacccgtgg	Verify $QcrA^{L349W}$

Supplementary Table 2. Primers used in cloning of the cytochrome bc expression vector and generation of point mutants.
